# The effect of sex steroid hormones on the ecology of *in vitro* oral biofilms

**DOI:** 10.1016/j.bioflm.2023.100139

**Published:** 2023-06-29

**Authors:** Pilar Cornejo Ulloa, Monique H. van der Veen, Bernd W. Brandt, Mark J. Buijs, Bastiaan P. Krom

**Affiliations:** Department of Preventive Dentistry, Academic Centre for Dentistry Amsterdam (ACTA), University of Amsterdam and VU University Amsterdam, Amsterdam, the Netherlands

**Keywords:** Oral biofilm, Sex steroid hormones, Proteolytic activity

## Abstract

Sex steroid hormones (SSH) such as oestrogen, progesterone and testosterone are cholesterol derived molecules that regulate various physiological processes. They are present in both blood and saliva, where they come in contact with oral tissues and oral microorganisms. Several studies have confirmed the effect of these hormones on different periodontal-disease-associated bacteria, using single-species models. Bacteria can metabolize SSH, use them as alternative for vitamin K and also use them to induce the expression of virulence factors. However, it is still unclear what the effects of SSH are on the oral microbiome. In this study, we investigated the effects of four SSH on commensal *in vitro* oral biofilms. Saliva-derived oral biofilms were grown in Mc Bain medium without serum or menadione using the Amsterdam Active-Attachment model. After initial attachment in absence of SSH, the biofilms were grown in medium containing either oestradiol, oestriol, progesterone or testosterone at a 100-fold physiological concentration. Menadione or ethanol were included as positive control and negative control, respectively. After 12 days with daily medium refreshments, biofilm formation, biofilm red fluorescence and microbial composition were determined. The supernatants were tested for proteolytic activity using the Fluorescence Resonance Energy Transfer Analysis (FRET). No significant differences were found in biofilm formation, red fluorescence or microbial composition in any of the tested groups. Samples grown in presence of progesterone and oestradiol showed proteolytic activity comparable to biofilms supplemented with menadione. In contrast, testosterone and oestriol showed a decreased proteolytic activity compared to biofilms grown in presence of menadione.

None of the tested SSH had large effects on the ecology of *in vitro* oral biofilms, therefore a direct translation of our results into *in vivo* effects is not possible. Future experiments should include other host factors such as oral tissues, immune cells and combinations of SSH as present in saliva, in order to have a more accurate picture of the phenomena taking place in both males and females.

## Graphical abstract

**Figure:**
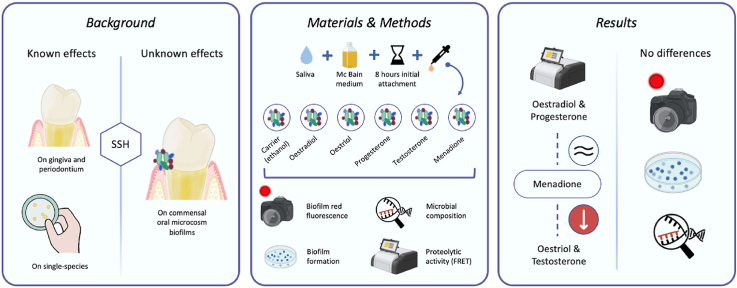

## 1 Introduction

Sex steroid hormones (SSH) are cholesterol-derived molecules, mostly secreted by the testes and the ovaries, and are responsible for a variety of physiological processes in the human body [1,2]. These hormones are known for their role in sexual development and reproduction. However, they are extremely versatile in their functions and are involved in a wide range of non-reproductive processes. For example, they are capable of influencing bone and tissue metabolism, cardiovascular and brain function, the immune response, amongst others [[3], [4], [5], [6], [7]].

After being secreted into the bloodstream, SSH reach different target tissues, including the oral tissues and the periodontium. SSH diffuse into the salivary glands’ acinar cells to then reach the oral cavity [8].

During periods of hormonal fluctuations such as puberty and pregnancy SSH trigger various responses, including inflammation and proliferation of cells [9,10]. These responses result in gingival inflammation, also known as gingivitis [11,12]. However, not only target tissues are influenced by the presence of SSH. Bacteria and other microorganisms living in and on the body also encounter SSH in their habitat and the oral cavity is no exception [9,13,14].

Several *in vitro* studies have shown that mainly —but not exclusively— periodontal-disease-associated bacteria can respond to the presence of SSH. In particular, three groups of SSH and their derivates, namely: progestogens, oestrogens and testosterone [13] can modify bacterial metabolism, growth and expression of virulence factors [[15], [16], [17]]. A characteristic virulence factor of several periodontal-disease-associated bacteria is their ability to produce protein-degrading enzymes (proteases) [18] which play a crucial role in the development of periodontal disease [19].

The effects of SSH on bacteria and yeast have been studied in several body niches, such as the gut [20] vagina [21] and respiratory tract [22], both *in vitro* and *in vivo.*

In the mouth, the effects of SSH on bacteria have been studied mostly in single-species models on periodontal-disease-associated bacteria. The reason behind this is based on the premise that periodontal-disease-associated bacteria can use SSH to replace a vital component to their growth: vitamin K [[16], [17], [18], [19], [20], [21], [22], [23]] observed that oestrogen and progesterone could compensate for the absence of vitamin K in the medium and thus support the viability and growth of the periodontal pathogens *Porphyromonas gingivalis* and *Prevotella melaninogenica*, amongst other periodontal-disease-associated bacteria. Since then, many *in vitro* studies have assessed different aspects of the interactions between SSH and oral bacteria [13].

*In vivo* fluctuations of SSH, both intrinsic (puberty, menstrual cycle and pregnancy) and extrinsic (use of artificial hormones), can lead to clinical and microbiological changes in the oral cavity [9,[24], [25], [26], [27], [28]]. Results from studies focusing on microbiological changes of specific bacteria and studies using broader approaches such as 16S rRNA gene amplicon sequencing suggest that hormonal shifts are associated with changes in the oral tissues and the oral microbiome. Interestingly, these changes are not limited to periodontal-disease-associated bacteria. Several studies have also reported interactions between SSH and caries associated bacteria, health associated bacteria and oral yeasts [13].

The effects of SSH on oral health result from the complex interaction between the host and its microbiome. In the present study we focus on the effects of SSH on the oral microbiota using a multi-species *in vitro* approach. This could help better understand the pathophysiology of non-dental plaque induced gingival disease. The aim of this *in vitro* study was to test the individual effects of oestradiol, oestriol, progesterone and testosterone on the growth, composition and metabolism of commensal oral microcosm biofilms.

## 2 Materials & methods

### 2.1 Saliva collection and storage

In analogy with previous studies [29], saliva was collected from two self-reported healthy donors (1 female, 1 male), following the unstimulated saliva spitting method protocol [30]. Donors refrained from dental hygiene for at least 24 h and did not eat or drink for 2 h before the collection. The collected saliva was pooled, mixed 1:1 with 60% sterile glycerol and aliquoted. The study was approved by the ACTA Ethical Committee (protocol number: 2021-40362).

### 2.2 Biofilm growth

Two independent experiments with four samples per condition were carried out using the Amsterdam Active Attachment model (AAA-model) [31]. Models were mounted with 12 mm sterile glass coverslips (Menzel, Braunschweig, Germany). Saliva-derived microcosm biofilms resembling commensal oral biofilms were grown as previously described [32]. Previously collected unstimulated saliva was thawed on ice and diluted 1:50 in McBain medium (2.5 g/l porcine mucin (Sigma, St Louis, Missouri), 2.0 g/l Bacto peptone (Difco, Detroit, Michigan), 2.0 g/l trypticase peptone (BBL, Cockeysville, Maryland), 1.0 g/l yeast extract (BD Diagnostic Systems, Sparks, Maryland), 0.35 g/l NaCl, 0.2 g/l KCl, 0.2 g/l CaCl_2_ and 1 mg/l hemin (Sigma, St. Louis, Missouri, USA) set at pH 7.0 [33]. For each replicate, a sample of the used inoculum was stored at −80 °C for DNA extraction. 1.5 ml of the inoculum was transferred to wells of a 24-well plate (CELLSTAR, Greiner Bio-one, Kremsmünster, Austria). The lid holding the coverslips was placed on the 24-well plate with the coverslips submerged in the inoculum. The models were stored in anaerobic jars (gas mix 10% CO_2_, 10% H_2_, and 80% N_2_) at 37 °C. After the 8-h inoculation period, the lid was placed on a new 24-well plate containing fresh medium supplemented with either one of the following components: (1) Menadione 0.01% w/v; (2) absolute ethanol (carrier) 0.01% v/v; (3) Oestradiol (E1024 Sigma-Aldrich, MO, USA) 10.9 ng/ml; (4) Oestriol (E1253 Sigma-Aldrich, MO, USA) 5 ng/ml; (5) Progesterone (P0130 Sigma-Aldrich, MO, USA) 2 μg/ml and; (6) Testosterone (86500 Sigma-Aldrich, MO, USA) 0.58 μg/ml. All hormones were dissolved in absolute ethanol and the final concentration was equivalent to 100-fold the physiological concentration in serum [[34], [35], [36], [37]]. The medium was refreshed every day for 11 days. On days 4, 8 and 12 the spent medium was collected from the wells and centrifuged for 10 min at 21,300×*g* at 4 °C. The supernatant was stored at −80 °C for further analysis. On day 12, the samples were harvested and different analyses were performed.

### 2.3 Red fluorescence of biofilms

Prior to harvesting, red fluorescence of the biofilms was determined [29]. The lid containing the biofilms was inverted and photographed in a dark room using a QLF-D camera system (Inspektor Research Systems BV, Amsterdam, the Netherlands) via image capture software (C31.25 Inspektor Research Systems BV, Amsterdam, the Netherlands). Red fluorescence was assessed qualitatively as presence or absence. It is a typical characteristic of anaerobic mature oral biofilms to fluoresce red [38]. This is due to their ability to accumulate porphyrins [39].

### 2.4 Harvest of the biofilms

To harvest the biofilms, all the glass coverslips were aseptically removed from the lid and transferred to sterile tubes containing 2 ml of phosphate buffered saline (8.0 g/l NaCl, 0.2 g/l KCl, 1.0 g/l Na_2_HPO_4_, 0.2 g/l KH_2_PO_4_) set at 7.4 pH. Biofilms were dislodged from the glass by sonication using a Vibracell VCX130 sonicator with a maximum of 130 W and 20 kHz for 45 s, with a pulse rate of 50% and pulses of 1 s at a vibration amplitude of 40% (Sonics & Materials, Newtown, USA). Samples were kept on ice to prevent heating.

### 2.5 Biofilm viability

To quantify biofilm viability, the number of anaerobic Colony Forming Units (CFU) of each biofilm was determined. Serial dilutions of the dispersed biofilms were plated on tryptic soy blood agar (TSBA) plates using an Eddy jet spiral plater (Neu-tec Group Inc., Farmingdale, NY). Plates were incubated anaerobically at 37 °C for 7 days and CFUs were counted. The remaining undiluted dispersed biofilms were centrifuged for 10 min at 21,300×*g* at 4 °C. Supernatants were discarded and the pellets were stored at −80 °C for DNA isolation and 16S rRNA gene amplicon sequencing.

### 2.6 Spent medium pH assessment

The pH of the medium after growth was determined using an iridium microelectrode (Beetrode MEPH-1, WPI Instruments, New Haven, Conn, USA) connected to a daily calibrated Orion SA720 pH/ISE Meter (Orion Research, Boston, Mass, USA).

### 2.7 Protease activity using a fluorescence resonance energy transfer analysis (FRET)

Protease activity in the spent medium was determined using the FRET analysis as described previously [40]. Two probes were used: (1) PEK-054 to detect unspecific increased proteolytic activity and (2) BikKam-15 [40] to detect specific *P. gingivalis* increased proteolytic activity. For this, 100 μl of Tris Buffered Saline (TBS: 50 mM Tris and 150 mM NaCl, pH 7.6) were added to each well of a Blackwell clear-bottom 96-well plate (Corning, Lowell, MA). Subsequently, 100 μl of the filtered culture supernatants were added in duplicates. Lastly, 4 μl of the selected FRET-probe (800 μM) were added to each well and the fluorescence was assessed immediately. Fluorescence intensity (excitation: 485 nm, emission: 530 nm) was measured every 5 min for 120 min at 37 °C using a SpectraMax i3x microplate reader (Molecular Devices, San Jose, CA). The protease activity was calculated from the initial linear slope and defined in Relative Fluorescence Units (RFU) per minute (RFU/min). All the values were then transformed to values relative to the carrier control, which was set at 100%.

### 2.8 DNA isolation

DNA isolation from dislodged biofilms was performed as described previously [41]. Briefly, biofilm pellets were thawed, resuspended with 150 μl Tris EDTA buffer (10 mM Tris-Cl (pH 8.0), 1 mM EDTA (pH 8.0)) and transferred to a well in a 96 deep well plate (Axygen Scientific Inc., CA, USA). As controls, the original inoculum and sterile McBain medium without additions from each experiment were included.

To each well, the following compounds were added: 250 μl of 0.1-mm diameter Zirconia beads (BioSpec Products, Bartlesville, OK, USA), 200 μl of phenol (Rotiphenol, Carl Roth GMBH&Co. KG, Germany) and 200 μl of lysis buffer (MagMini DNA isolation kit, LGC Genomics Ltd, UK). The plate was then sealed and placed in a Mini-BeadBeater-96 (BioSpec Products, Bartlesville, OK, USA) for 2 min at 2.100 oscillations/min. When this process was completed, DNA was extracted and purified with the MagMini DNA Isolation Kit (MagMini DNA isolation kit, LGC Genomics Ltd, UK). Bacterial DNA concentration was determined by qPCR as described elsewhere [42].

### 2.9 16S rRNA gene amplicon sequencing and data processing

To assess the bacterial composition of the biofilms exposed to the different hormones, DNA was further processed for sequencing as described above. For this, the V4 hypervariable region of the 16S rRNA gene was used and the equimolar mix was sequenced using the Illumina MiSeq platform (Core Facility Genomics, AmsterdamUMC, The Netherlands). The paired-end reads were quality-filtered, merged and clustered into operational taxonomic units (OTUs) at 97% similarity as described by Koopman et al. [43]. The most abundant sequence of each OTU was assigned a taxonomy using the Ribosomal Database Project (RDP) classifier [44] implemented in QIIME 1.9.1 and the SILVA database (version 132, [45]). The OTU table was subsampled at a depth of 13,950 reads per sample to allow comparisons among the samples.

### 2.10 Statistical analyses

One-way ANOVA and the Bonferroni post-hoc test were performed to analyse the total cultivatable cell counts. Results derived from the FRET analysis were assessed using ANOVA and no post-hoc correction was used [46]. Maximal activity, represented by RFU/min was analysed. Differences were considered statistically significant if *p* < 0.05.

The Shannon diversity index (unbiased version) and observed OTUs (richness), calculated using PAST v4.10 [47] were used to characterize the α-diversity. Next, the Kruskal-Wallis test and Dunn's test for multiple comparisons were performed using GraphPad v9.4.1 for Windows (GraphPad Software, San Diego, California USA, www.graphpad.com). The log2-transformed sequencing data (OTU table) was ordinated using a Principal-Component Analysis (PCA) to compare two groups of experimental runs. Statistical differences in the microbial profiles were analysed with one-way Permutational Multivariate Analysis of Variance (PERMANOVA), using the Bray-Curtis distance and 9999 permutations, to identify possible differences in microbiological profiles between the different treatment conditions (Menadione, Ethanol (carrier), Oestradiol, Oestriol, Progesterone and Testosterone). The latter analyses were performed using R version 4.1.3 [48] and RStudio (RStudio: Integrated Development for R. RStudio, PBC, Boston, MA). Differences were considered statistically significant if *p* < 0.05.

The metagenomic biomarker discovery tool Linear discriminant analysis Effect Size (LefSe) [49] was used to identify microbial taxa that significantly differed between the treatment group (a hormone) versus ethanol (carrier control). This was done using a filtered OTU table where OTUs with ≤100 reads were not considered in the analysis. Default LEfSe settings were used. The online Galaxy platform (http://huttenhower.sph.harvard.edu/galaxy) was used to perform the LEfSe analyses.

## 3 Results

### 3.1 Red fluorescence, biofilm formation and pH

**Fig. 1:**
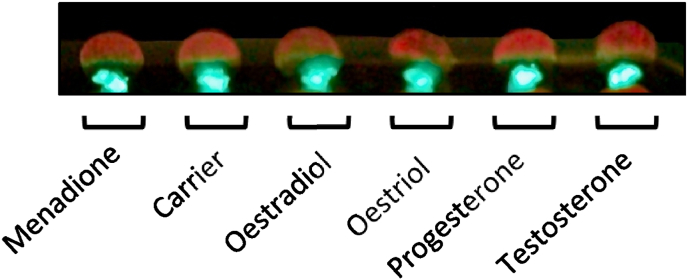
Red fluorescence photograph of biofilms grown on glass discs in the AAA-model. The glass discs were fixed with tweezers and fluid silicone, which fluoresces green. The biofilms located on the upper part of the discs, fluoresce red. For simplification purposes, only one disc per condition is shown on the photograph. From left to right, biofilms grown with added: menadione, absolute ethanol, oestradiol, oestriol, progesterone and testosterone. No differences were observed between the groups. (For interpretation of the references to colour in this figure legend, the reader is referred to the Web version of this article.)

**Fig. 2:**
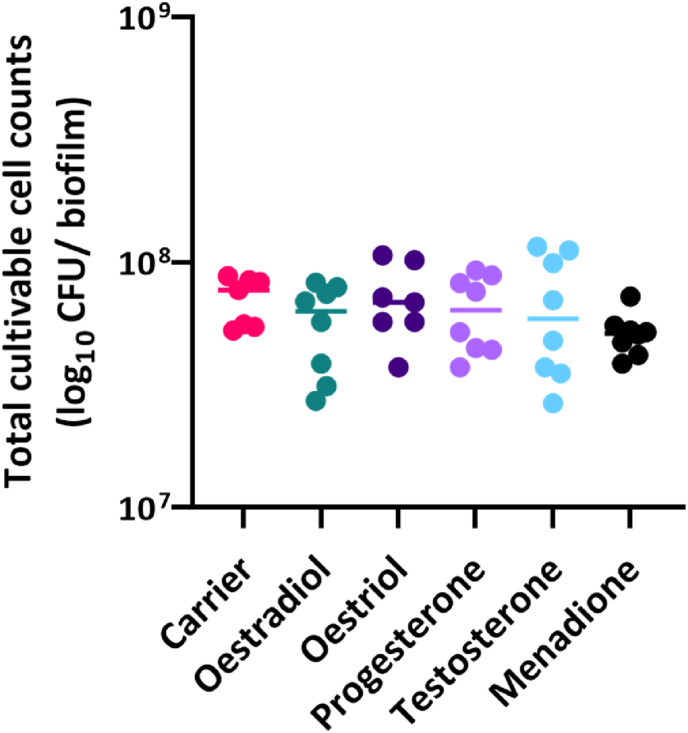
Quantification of biofilm viability in the presence of different SSH. Biofilm formation was estimated based on total viable cultivatable cell counts (CFU). Positive control: menadione (medium with added 0.01% menadione); Negative control: carrier (medium with added 0.01% absolute ethanol). Total cultivatable cell counts were ∼10^8^, with no differences between the groups. Two independent experiments were performed with four samples per condition.

In our experiment, no differences in the red fluorescence of the biofilms were observed (Fig. 1). The total cultivatable cell counts did not show any differences between the groups (Fig. 2). Similarly, the pH of the spent medium did not show any hormone-dependent differences (7.09 ± 0.25).

### 3.2 Effect of SSH on proteolytic activity

**Fig. 3:**
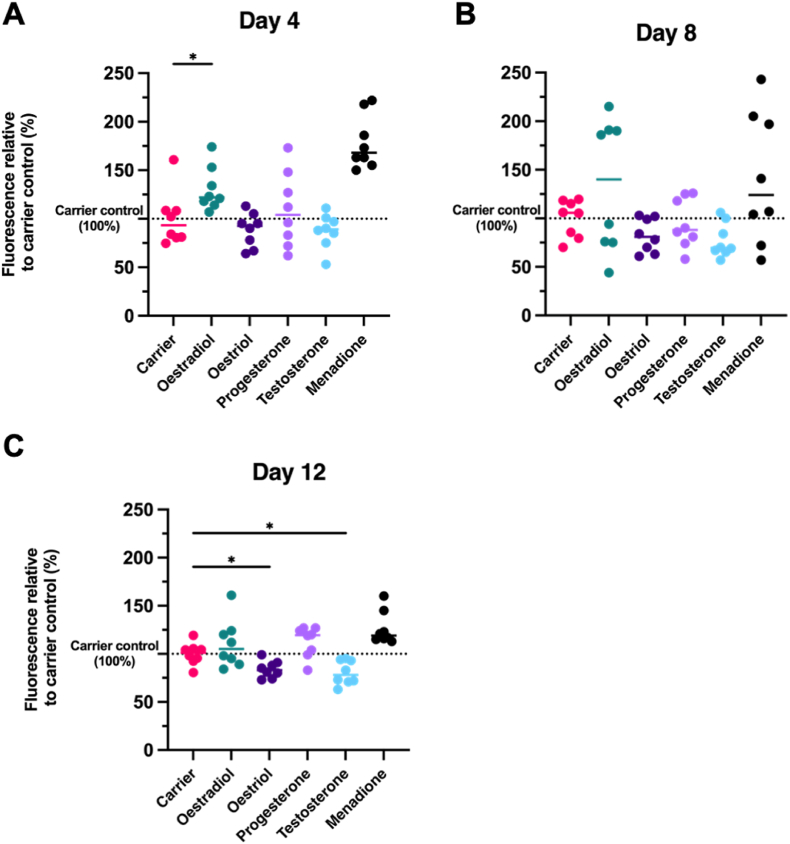
Unspecific proteolytic activity of the spent medium expressed in percentage relative Fluorescence Units relative to carrier (0.01% added absolute ethanol). Measurements were performed using FRET assay and PEK-054 probe. **A.** Proteolytic activity measured on biofilm's supernatant after 4 days **B.** Proteolytic activity measured on biofilm's supernatant after 8 days **C.** Proteolytic activity measured on biofilm's supernatant after 12 days. Significant differences (*p ≤ 0.05) can be seen on days 4 and 12 between the carrier and SSH. Results from day 8 were not statistically significant. Only comparisons between carrier and SSH are shown. Two independent experiments were performed with four samples per condition.

Differences between the groups were observed when assessing specific and unspecific proteolytic activity. Samples from days 4, 8 and 12 were tested. Specific *P. gingivalis* activity was not observed in any of the samples (data not shown). Unspecific proteolytic activity using the probe PEK-054 showed a separation in two groups on days 4, 8 and 12. The highest proteolytic activity was detected on the samples with added menadione, oestrogen and progesterone. The lowest proteolytic activity group was detected on the samples with added oestriol and testosterone (Fig. 3). This trend was the most distinct at day 12.

### 3.3 Relative abundance of the predominant bacterial genera and α-diversity

**Fig. 4:**
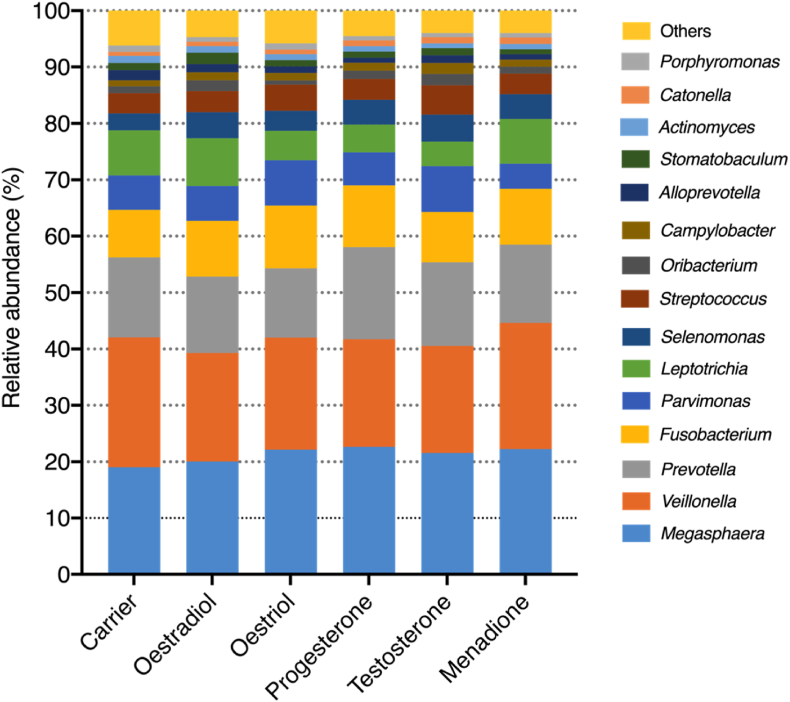
Taxonomy plot of the 15 most abundant genera or higher taxa in the biofilms treated with either: absolute ethanol, oestradiol, oestriol, progesterone, testosterone and menadione. The remaining genera are labelled as “others”. Two independent experiments were performed with four samples per condition.

The average relative abundance of the 15 most abundant bacterial genera for each condition are shown in Fig. 4. All conditions presented a similar composition despite the addition of different compounds. All samples were composed of approximately 20% *Megasphaera*, 20% *Veillonella*, 10% *Prevotella* and 50% of a variety of taxa including *Fusobacterium*, *Parvimonas*, *Leptotrichia* amongst others. A slight, but not significant difference, in the relative abundance of *Parvimonas* and *Leptotrichia* was observed in the samples with the lowest proteolytic activity (Oestriol and Testosterone). Specifically, *Parvimonas* showed a slight increase compared to the rest of the conditions whereas *Leptotrichia* showed a slight decrease.

**Fig. 5:**
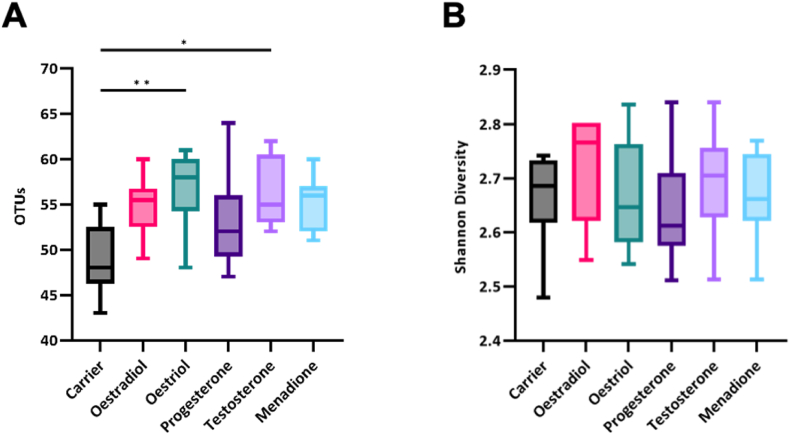
Boxplots showing the α-diversity of the different biofilms supplemented with either absolute ethanol, oestradiol, oestriol, progesterone, testosterone and menadione. **A.** Number of observed OTUs in the different groups with significant differences between the different conditions are indicated (Kruskal-Wallis: p = 0.0133). **B.** Shannon diversity index, no differences between groups. Two independent experiments were performed with four samples per condition.

Both richness –estimated by Operational Taxonomic Units (OTUs)– and Shannon diversity were calculated to assess α-diversity (Fig. 5). The number of OTUs in the inoculum was 106 (data not shown). The number of OTUs in all samples was 54 ± 10. Comparing the number of OTUs between the different conditions, oestriol (p = 0.0145) and testosterone (p = 0.045) exhibited a significantly higher diversity as compared to the negative control (carrier) (Fig. 5a). The Shannon diversity index did not show any significant differences between the groups (Fig. 5b).

### 3.4 Microbial composition of the microcosm biofilms

**Fig. 6:**
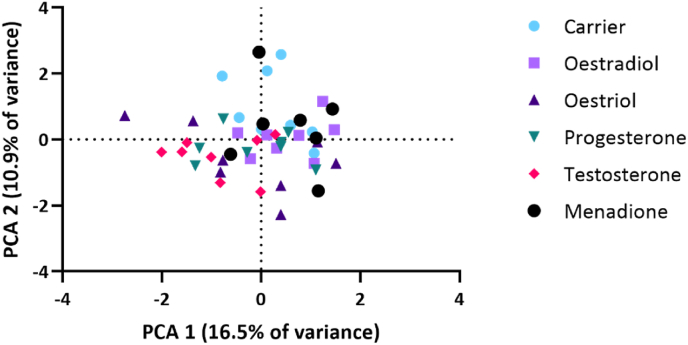
Principal Component Analysis (PCA) plot of the biofilms treated with the different compounds. No specific clustering pattern can be observed for each individual condition. Two independent experiments were performed with four samples per condition.

To investigate possible microbial composition differences in the biofilms induced by SSH, bacterial composition of the biofilms was determined using 16S rRNA gene sequencing. The microbial profiles were plotted using a Principal Component Analysis (PCA) (Fig. 6). No obvious clustering of samples for each treatment was detected. Permutational Multivariate Analysis of Variance analysis (PERMANOVA) showed significant differences in composition only between the biofilms exposed to ethanol (carrier) and testosterone (p = 0.026). The differences between the biofilms exposed to menadione and oestriol approached the cut-off value for statistical significance (p = 0.055). Accordingly, linear discriminant analysis effect size (LEfSe) did not show any significant differences in specific bacteria between the tested conditions and ethanol (carrier control).

### 3.5 Reproducibility of the experiments

To evaluate the inter and intra experiment reproducibility, the Bray-Curtis similarity indices were averaged within a batch and between the two experimental batches, for each condition. Both experiments showed an intra experiment similarity (range: 0.79–0.86, 0.83–0.86 resp.), and high inter experiment similarity (range: 0.82–0.85) indicating that the experimental approach is highly reproducible.

## 4 Discussion

In the present study, we assessed the response of an *in vitro* oral microcosm to the presence of oestradiol, oestriol, progesterone and testosterone. The response of the biofilms to these compounds did not differ significantly on most of the tested parameters. However, biofilms grown in the presence of oestradiol and testosterone showed a lower proteolytic activity and a higher microbial diversity (OTUs) than the other SSH. All things considered, none of the tested SSH had large effects on either the growth or composition of *in vitro* oral biofilms, therefore a direct translation of our results into *in vivo* effects is not possible.

The aim of the study was to assess possible changes in a healthy commensal microcosm, similar to the study by Janus et al. [32]. For this reason, saliva was selected as the inoculum. Saliva can be collected in an easy and non-invasive manner. Also, saliva is regarded as a fair representation of several niches in the oral cavity [50].

Several *in vitro* studies have assessed the effects of SSH on single-species models, with positive results [13]. Mostly periodontal-disease-associated bacteria have been studied with several reports documenting their ability to use SSH to support different biological processes [[15], [16], [17]]. Therefore, it is remarkable that in the present study: (1) a very limited effect of SSH could be measured in commensal oral microcosm biofilms and; (2) there was no clear selection of periodontal-disease-associated bacteria as determined by 16S rDNA sequencing.

It is unexpected that both menadione and hormones did not show large differences in our model system. The experimental design was aimed to test the exclusive effect of SSH on commensal oral biofilms. For this reason, menadione was used as a positive control as it is known to be vital for the growth of certain periodontal-disease-associated bacteria [51]. In our experiment, menadione did not specifically support the growth of these bacterial species (Fig. 4). This suggests that the addition of menadione does not significantly influence the composition of a multi-species commensal oral biofilm as would be expected based on single-species models.

This could be explained by previous studies that have evaluated the synthesis of vitamin K by specific microbes. Ibrahim et al. [52] reported that oral *Prevotella* spp. can synthesize vitamin K from chorismite, with the help of MenA-MenG enzymes (classical menaquinone pathway). These enzymes have also been identified in *Megasphaera* spp*.* [53]*, V. parvula* [54], *F. nucleatum* [55], *Actinomyces* [56], and *Porphyromonas* [[57], [58], [59]]. Also, *C. rectus* is capable of producing vitamin K from chorismite via de Futalosine pathway by action of MqnABCD enzymes [60]. The aforementioned genera were present in our samples (Fig. 4). Several of these genera have been observed in another study using a similar methodology [61]. The absence of difference in composition between biofilms supplemented with menadione and the carrier control suggests that bacteria present in oral microcosm biofilms are indeed capable of producing vitamin K.

While we did not detect differences in microbial composition of oral biofilms grown in the presence of different SSH, significant differences in proteolytic activity of these biofilms were observed. When measuring proteolytic activity, samples supplemented with a known analogue for vitamin K (oestrogen and progesterone) showed a level of proteolytic activity comparable to the one exhibited by biofilms supplemented with menadione. In contrast, addition of oestriol and testosterone resulted in a significantly lower proteolytic activity. This suggests that oestriol and testosterone may not function as substitutes for vitamin K.

The observed difference in proteolytic activity could not be explained based on the microbial composition. However, differences in enzyme activity might be related to differences in expression. For instance, *Parvimonas micra* has been reported to stimulate the expression of the virulence factor gingipains of *P. gingivalis* [62] In our study, we did not evaluate the effects of SSH on gene expression in biofilms. For now, we can only conclude that the presence of oestradiol and progesterone induced a small yet significant increase of proteolytic enzymes similar to menadione. On the contrary, oestriol and testosterone induced a decrease in the presence of these enzymes.

Periodontal diseases such as gingivitis and periodontitis are often characterised by the presence of dysbiotic biofilms [[63], [64], [65]]. Also, periodontal-disease-associated bacteria are known to be more proteolytic and commonly produce proteases [66,67]. These proteases have been recently clinically measured using a FRET-based assay [68]. Bikker et al. reported an increase of salivary proteolytic activity on subjects taking part in an induced-gingivitis clinical study. This increased proteolytic activity could be of key importance to assess a patient's risk of developing periodontal disease. Although the *in vitro* differences were statistically significant, we should be careful to directly translate these results to an *in vivo* situation.

There is consensus that sex steroid hormones can cause morphological and metabolic changes of the gingival tissues during periods of hormone surges [9,69]. Direct changes in oral biofilms by hormonal changes though, are understudied and remain controversial. Changes in periodontal-disease-associated bacteria have been reported during puberty [28], the menstrual cycle [25], pregnancy [11,24,70,71], on women with hormonal disorders [72] and by users of synthetic SSH [73]. These reports all have something in common: changes in periodontal-disease-associated bacteria occur during periods of hormonal fluctuations. Taking into consideration that there might be an adaptation of the oral microbiome to the constant presence of SSH *in vivo*, distortion of the regular balance could explain compositional changes. To test this in an *in vitro* model, a longer exposure time would be needed along with changes in the concentration of added SSH to mimic hormonal fluctuations.

Also, several studies report a sex-preference in the incidence of periodontitis, with men being more susceptible than women [74]. However, based on our *in vitro* study this difference cannot be associated to distinct sex-specific-SSH effects on the oral microbiota. The absence of SSH-induced effects suggests that SSH alone are not responsible for changes in the oral microbiome *in vivo.* SSH play multiple roles including in the physiology of the host by modulating the immune system [[75], [76], [77]]. It is certainly possible that this host response to SSH modulates the microbiome to a larger extent than the direct modulation of the microbiome by SSH.

The complex crosstalk between the oral microbiome, SSH and oral tissues include many different factors that influence the microbiome and host's response in ways that have yet to be explained. In our *in vitro* model, SSH differentially modulated the proteolytic activity of oral biofilms. To our knowledge, there are no other studies that have tested this on an *in vitro* oral microcosm. Future experiments should include other host factors such as oral tissues, immune cells and combinations of SSH as present in saliva, in order to have a more accurate picture of the phenomena taking place in both males and females.

This research did not receive any specific grant from funding agencies in the public, commercial, or not-for-profit sectors.

## CRediT authorship contribution statement

**Pilar Cornejo Ulloa:** Conceptualization, Methodology, Validation, Formal analysis, Investigation, Writing – original draft, Visualization. **Monique H. van der Veen:** Conceptualization, Methodology, Validation, Writing – review & editing, Supervision, Project administration. **Bernd W. Brandt:** Software, Validation, Formal analysis, Data curation, Writing – review & editing. **Mark J. Buijs:** Formal analysis, Writing – review & editing. **Bastiaan P. Krom:** Conceptualization, Methodology, Validation, Writing – review & editing, Supervision, Project administration.

## Declaration of competing interest

The authors declare the following financial interests/personal relationships which may be considered as potential competing interests: Corresponding author (Bastiaan P. Krom) is serving in an editorial capacity for the journal we are submitting to

## Data Availability

Data will be made available on request.

## References

[1] Chan L., O'Malley B.W. (1976). Mechanism of action of the sex steroid hormones (first of three parts). N Engl J Med.

[2] Hu J., Zhang Z., Shen W.-J., Azhar S. (2010). Cellular cholesterol delivery, intracellular processing and utilization for biosynthesis of steroid hormones. Nutr Metabol.

[3] Baños G., Guarner V., Pérez-Torres I. (2011). Sex steroid hormones, cardiovascular diseases and the metabolic syndrome. Cardiovasc Hematol Agents Med Chem.

[4] Garcia-Gomez E., Gonzalez-Pedrajo B., Camacho-Arroyo I. (2013). Role of sex steroid hormones in bacterial-host interactions. BioMed Res Int.

[5] Pompili A., Iorio C., Gasbarri A. (2020). Effects of sex steroid hormones on memory. Acta Neurobiol Exp.

[6] Ruggieri A., Anticoli S., D'Ambrosio A., Giordani L., Viora M. (2016). The influence of sex and gender on immunity, infection and vaccination. Ann Ist Super Sanita.

[7] Xu K., Fu Y., Cao B., Zhao M. (2022). Association of sex hormones and sex hormone-binding globulin levels with bone mineral density in adolescents aged 12-19 years. Front Endocrinol.

[8] Vining R.F., McGinley R.A., Symons R.G. (1983). Hormones in saliva: mode of entry and consequent implications for clinical interpretation. Clin Chem.

[9] Mariotti A., Mawhinney M. (2013). Endocrinology of sex steroid hormones and cell dynamics in the periodontium. Periodontol.

[10] Markou E., Eleana B., Lazaros T., Antonios K. (2009). The influence of sex steroid hormones on gingiva of women. Open Dent J.

[11] Kumar P.S. (2013). Sex and the subgingival microbiome: do female sex steroids affect periodontal bacteria?. Periodontol.

[12] Mealey B.L., Moritz A.J. (2003). Hormonal influences: effects of diabetes mellitus and endogenous female sex steroid hormones on the periodontium. Periodontol.

[13] Cornejo Ulloa P., Krom B.P., van der Veen M.H. (2021). Sex steroid hormones as a balancing factor in oral host microbiome interactions. Front Cell Infect Microbiol.

[14] Vom Steeg L.G., Klein S.L. (2017). Sex steroids mediate bidirectional interactions between hosts and microbes. Horm Behav.

[15] Fteita D., Kononen E., Soderling E., Gursoy U.K. (2014). Effect of estradiol on planktonic growth, coaggregation, and biofilm formation of the Prevotella intermedia group bacteria. Anaerobe.

[16] Kornman K.S., Loesche W.J. (1982). Effects of estradiol and progesterone on Bacteroides melaninogenicus and Bacteroides gingivalis. Infect Immun.

[17] Yokoyama M., Hinode D., Masuda K., Yoshioka M., Grenier D. (2005). Effect of female sex hormones on Campylobacter rectus and human gingival fibroblasts. Oral Microbiol Immunol.

[18] Potempa J., Banbula A., Travis J. (2000). Role of bacterial proteinases in matrix destruction and modulation of host responses. Periodontol.

[19] Potempa J., Pike R.N. (2009). Corruption of innate immunity by bacterial proteases. J Innate Immun.

[20] Baker J.M., Al-Nakkash L., Herbst-Kralovetz M.M. (2017). Estrogen-gut microbiome axis: physiological and clinical implications. Maturitas.

[21] Kwon M.S., Lee H.K. (2022). Host and microbiome interplay shapes the vaginal microenvironment. Front Immunol.

[22] Chotirmall S.H., Smith S.G., Gunaratnam C., Cosgrove S., Dimitrov B.D., O'Neill S.J., McElvaney N.G. (2012). Effect of estrogen on pseudomonas mucoidy and exacerbations in cystic fibrosis. N Engl J Med.

[23] Gibbons R.J., Macdonald J.B. (1960). Hemin and vitamin K compounds as required factors for the cultivation of certain strains of Bacteroides melaninogenicus. J Bacteriol.

[24] Balan P., Chong Y.S., Umashankar S., Swarup S., Loke W.M., Lopez V., Seneviratne C.J. (2018). Keystone species in pregnancy gingivitis: a snapshot of oral microbiome during pregnancy and postpartum period. Front Microbiol.

[25] Bostanci N., Krog M.C., Hugerth L.W., Bashir Z., Fransson E., Boulund F., Schuppe-Koistinen I. (2021). Dysbiosis of the human oral microbiome during the menstrual cycle and vulnerability to the external exposures of smoking and dietary sugar. Front Cell Infect Microbiol.

[26] Brusca, Verdugo F., Amighini C., Albaina O., Moragues M.D. (2014). Anabolic steroids affect human periodontal health and microbiota. Clin Oral Invest.

[27] Kornman K.S., Loesche W.J. (1980). The subgingival microbial flora during pregnancy. J Periodontal Res.

[28] Wojcicki C.J., Harper D.S., Robinson P.J. (1987). Differences in periodontal disease-associated microorganisms of subgingival plaque in prepubertal, pubertal and postpubertal children. J Periodontol.

[29] Janus M.M., Crielaard W., Volgenant C.M., van der Veen M.H., Brandt B.W., Krom B.P. (2017). Candida albicans alters the bacterial microbiome of early in vitro oral biofilms. J Oral Microbiol.

[30] Navazesh M., Christensen C.M. (1982). A comparison of whole mouth resting and stimulated salivary measurement procedures. J Dent Res.

[31] Exterkate R.A., Crielaard W., Ten Cate J.M. (2010). Different response to amine fluoride by Streptococcus mutans and polymicrobial biofilms in a novel high-throughput active attachment model. Caries Res.

[32] Janus M.M., Keijser B.J., Bikker F.J., Exterkate R.A., Crielaard W., Krom B.P. (2015). In vitro phenotypic differentiation towards commensal and pathogenic oral biofilms. Biofouling.

[33] McBain A.J., Sissons C., Ledder R.G., Sreenivasan P.K., De Vizio W., Gilbert P. (2005). Development and characterization of a simple perfused oral microcosm. J Appl Microbiol.

[34] England P.C., Skinner L.G., Cottrell K.M., Sellwood R.A. (1974). Serum oestradiol-17 beta in normal women. Br J Cancer.

[35] Gao W.L., Wu L.S., Zi J.H., Wu B., Li Y.Z., Song Y.C., Cai D.Z. (2015). Measurement of serum estrogen and estrogen metabolites in pre- and postmenopausal women with osteoarthritis using high-performance liquid chromatography-electrospray ionization-tandem mass spectrometry. Braz J Med Biol Res.

[36] Tajar A., Huhtaniemi I.T., O'Neill T.W., Finn J.D., Pye S.R., Lee D.M., Wu F.C. (2012). Characteristics of androgen deficiency in late-onset hypogonadism: results from the European Male Aging Study (EMAS). J Clin Endocrinol Metab.

[37] Taraborrelli S. (2015). Physiology, production and action of progesterone. Acta Obstet Gynecol Scand.

[38] Kim Y.-S., Lee E.-S., Kwon H.-K., Kim B.-I. (2014). Monitoring the maturation process of a dental microcosm biofilm using the Quantitative Light-induced Fluorescence-Digital (QLF-D). J Dent.

[39] Volgenant C.M., van der Veen M.H., de Soet J.J., ten Cate J.M. (2013). Effect of metalloporphyrins on red autofluorescence from oral bacteria. Eur J Oral Sci.

[40] Kaman W.E., Galassi F., de Soet J.J., Bizzarro S., Loos B.G., Veerman E.C., Bikker F.J. (2012). Highly specific protease-based approach for detection of porphyromonas gingivalis in diagnosis of periodontitis. J Clin Microbiol.

[41] Kahharova D., Brandt B.W., Buijs M.J., Peters M., Jackson R., Eckert G., Zaura E. (2020). Maturation of the oral microbiome in caries-free toddlers: a longitudinal study. J Dent Res.

[42] Ciric L., Pratten J., Wilson M., Spratt D. (2010). Development of a novel multi-triplex qPCR method for the assessment of bacterial community structure in oral populations. Environ Microbiol Rep.

[43] Koopman J.E., Buijs M.J., Brandt B.W., Keijser B.J., Crielaard W., Zaura E. (2016). Nitrate and the origin of saliva influence composition and short chain fatty acid production of oral microcosms. Microb Ecol.

[44] Wang Q., Garrity G.M., Tiedje J.M., Cole J.R. (2007). Naive Bayesian classifier for rapid assignment of rRNA sequences into the new bacterial taxonomy. Appl Environ Microbiol.

[45] Quast C., Pruesse E., Yilmaz P., Gerken J., Schweer T., Yarza P., Glöckner F.O. (2013). The SILVA ribosomal RNA gene database project: improved data processing and web-based tools. Nucleic Acids Res.

[46] Parker R.A., Weir C.J. (2020). Non-adjustment for multiple testing in multi-arm trials of distinct treatments: rationale and justification. Clin Trials.

[47] Hammer Ø., Harper D.A.T., Ryan P.D. (2001). PAST: paleontological Statistics software package for education and data analysis (Version 4.10). Palaeontol Electron.

[48] R Core Team (2022). https://www.R-project.org/.

[49] Segata N., Izard J., Waldron L., Gevers D., Miropolsky L., Garrett W.S., Huttenhower C. (2011). Metagenomic biomarker discovery and explanation. Genome Biol.

[50] Krishnan K., Chen T., Paster B. (2017). A practical guide to the oral microbiome and its relation to health and disease. Oral Dis.

[51] Mayrand D., Holt S.C. (1988). Biology of asaccharolytic black-pigmented Bacteroides species. Microbiol Rev.

[52] Ibrahim M., Subramanian A., Anishetty S. (2017). Comparative pan genome analysis of oral Prevotella species implicated in periodontitis. Funct Integr Genomics.

[53] Marx H., Graf A.B., Tatto N.E., Thallinger G.G., Mattanovich D., Sauer M. (2011). Genome sequence of the ruminal bacterium Megasphaera elsdenii. J Bacteriol.

[54] Gronow S., Welnitz S., Lapidus A., Nolan M., Ivanova N., Glavina Del Rio T., Lucas S. (2010). Complete genome sequence of Veillonella parvula type strain (Te3). Stand Genomic Sci.

[55] Kapatral V., Anderson I., Ivanova N., Reznik G., Los T., Lykidis A., Overbeek R. (2002). Genome sequence and analysis of the oral bacterium *Fusobacterium nucleatum* strain ATCC 25586. J Bacteriol.

[56] Holder M.E., Ajami N.J., Petrosino J.F. (2015). https://www.ncbi.nlm.nih.gov/protein/ALC99810.

[57] Naito M., Hirakawa H., Yamashita A., Ohara N., Shoji M., Yukitake H., Nakayama K. (2008). Determination of the genome sequence of porphyromonas gingivalis strain ATCC 33277 and genomic comparison with strain W83 revealed extensive genome rearrangements in P. Gingivalis. DNA Res.

[58] Nelson K.E., Fleischmann R.D., DeBoy R.T., Paulsen I.T., Fouts D.E., Eisen J.A., Fraser C.M. (2003). Complete genome sequence of the oral pathogenic Bacterium porphyromonas gingivalis strain W83. J Bacteriol.

[59] Watanabe T., Maruyama F., Nozawa T., Aoki A., Okano S., Shibata Y., Abiko Y. (2011). Complete genome sequence of the bacterium Porphyromonas gingivalis TDC60, which causes periodontal disease. J Bacteriol.

[60] Gemmell M.R., Berry S., Mukhopadhya I., Hansen R., Nielsen H.L., Bajaj-Elliott M., Hold G.L. (2018). Comparative genomics of Campylobacter concisus: analysis of clinical strains reveals genome diversity and pathogenic potential. Emerg Microb Infect.

[61] Janus M.M., Crielaard W., Zaura E., Keijser B.J., Brandt B.W., Krom B.P. (2016). A novel compound to maintain a healthy oral plaque ecology in vitro. J Oral Microbiol.

[62] Neilands J., Davies J.R., Bikker F.J., Svensäter G. (2019). Parvimonas micra stimulates expression of gingipains from Porphyromonas gingivalis in multi-species communities. Anaerobe.

[63] Caton J.G., Armitage G., Berglundh T., Chapple I.L.C., Jepsen S., Kornman K.S., Tonetti M.S. (2018). A new classification scheme for periodontal and peri-implant diseases and conditions – introduction and key changes from the 1999 classification. J Periodontol.

[64] Papapanou P.N., Sanz M., Buduneli N., Dietrich T., Feres M., Fine D.H., Tonetti M.S. (2018). Periodontitis: consensus report of workgroup 2 of the 2017 world workshop on the classification of periodontal and peri-implant diseases and conditions. J Periodontol.

[65] Trombelli L., Farina R., Silva C.O., Tatakis D.N. (2018). Plaque-induced gingivitis: case definition and diagnostic considerations. J Clin Periodontol.

[66] Byrne D.P., Manandhar S.P., Potempa J., Smalley J.W. (2015). Breakdown of albumin and haemalbumin by the cysteine protease interpain A, an albuminase of Prevotella intermedia. BMC Microbiol.

[67] Mallorquí-Fernández N., Manandhar S.P., Mallorquí-Fernández G., Usón I., Wawrzonek K., Kantyka T., Gomis-Rüth F.X. (2008). A new autocatalytic activation mechanism for cysteine proteases revealed by Prevotella intermedia interpain A. J Biol Chem.

[68] Bikker F.J., Nascimento G.G., Nazmi K., Silbereisen A., Belibasakis G.N., Kaman W.E., Bostanci N. (2019). Salivary total protease activity based on a broad-spectrum fluorescence resonance Energy transfer approach to monitor induction and resolution of gingival inflammation. Mol Diagn Ther.

[69] Gürsoy M., Zeidán-Chuliá F., Könönen E., Moreira J.C., Liukkonen J., Sorsa T., Gürsoy U.K. (2014). Pregnancy-induced gingivitis and OMICS in dentistry: in silico modeling and in vivo prospective validation of estradiol-modulated inflammatory biomarkers. OMICS.

[70] Gürsoy M., Haraldsson G., Hyvönen M., Sorsa T., Pajukanta R., Könönen E. (2009). Does the frequency of Prevotella intermedia increase during pregnancy?. Oral Microbiol Immunol.

[71] Machado F.C., Cesar D.E., Apolonio A.C., Ribeiro L.C., Ribeiro R.A. (2016). Longitudinal study on clinical and microbial analysis of periodontal status in pregnancy. Braz Oral Res.

[72] Akcalı A., Bostanci N., Özçaka Ö., Öztürk-Ceyhan B., Gümüş P., Buduneli N., Belibasakis G.N. (2014). Association between polycystic ovary syndrome, oral microbiota and systemic antibody responses. PLoS One.

[73] Tarkkila L., Kari K., Furuholm J., Tiitinen A., Meurman J.H. (2010). Periodontal disease-associated micro-organisms in peri-menopausal and post-menopausal women using or not using hormone replacement therapy. A two-year follow-up study. BMC Oral Health.

[74] Ioannidou E. (2017). The sex and gender intersection in chronic periodontitis. Front Public Health.

[75] Belkaid Y., Hand T.W. (2014). Role of the microbiota in immunity and inflammation. Cell.

[76] García-Gómez E., González-Pedrajo B., Camacho-Arroyo I. (2013). Role of sex steroid hormones in bacterial-host interactions. BioMed Res Int.

[77] Lapp C.A., Lapp D.F. (2005). Analysis of interleukin-activated human gingival fibroblasts: modulation of chemokine responses by female hormones. J Periodontol.

